# Supplementary material for: Regular Exercise Training Induces More Changes on Intestinal Glucose Uptake from Blood and Microbiota Composition in Leaner Compared to Heavier Individuals in Monozygotic Twins Discordant for BMI
Source: Nutrients. 2024 Oct 20;16(20):3554. doi: 10.3390/nu16203554 (PMC11510543; doi:10.3390/nu16203554)
Supplement: Supplementary file 1 [file nutrients-16-03554-s001.zip › nutrients-3247171-supplementary.pdf]

nutrients MDPI

Supplemental table S1. Type 1 analyses between microbiota, exercise and nutritional variables.

Megamonas					
Order	F-ratio	P-value	Order	F-ratio	P-value
Time	18.886	<.0001	Cho	0.000	0.99
Cho	0.596	0.44	Time	19.480	<.0001
Time	18.892	<.0001	sugar	0.489	0.49
sugar	0.608	0.44	Time	19.345	<.0001
Time	18.814	<.0001	Glucose	0.076	0.79
Glucose	0.285	0.60	Time	19.024	<.0001
Time	18.708	<.0001	Fructose	0.655	0.42
Fructose	0.084	0.77	Time	18.136	<.0001
Time	19.024	<.0001	Sucrose	0.320	0.57
Sucrose	0.771	0.38	Time	19.475	<.0001
Time	19.314	<.0001	D-fiber	2.577	0.11
D-fiber	0.281	0.60	Time	17.018	0.000
**Helicobacter**					
Order	F-ratio	P-value	Order	F-ratio	P-value
Time	19.773	<.0001	Cho	0.023	0.88
Cho	0.425	0.52	Time	22.176	<.0001
Time	19.834	<.0001	sugar	0.489	0.49
sugar	0.001	0.98	Time	19.345	<.0001
Time	19.696	<.0001	Glucose	0.967	0.33
Glucose	0.028	0.87	Time	18.768	<.0001
Time	20.036	<.0001	Fructose	2.787	0.10
Fructose	0.314	0.58	Time	17.563	0.000
Time	19.704	<.0001	Sucrose	0.053	0.82
Sucrose	0.007	0.93	Time	19.658	<.0001
Time	19.721	<.0001	D-fiber	0.359	0.55
D-fiber	0.033	0.86	Time	19.633	<.0001
**Sellimonas**					
Order	F-ratio	P-value	Order	F-ratio	P-value
Time	14.005	0.000	Cho	0.038	0.85
Cho	0.228	0.64	Time	14.195	0.000
Time	13.992	0.001	sugar	0.000	1.0
sugar	0.380	0.54	Time	14.372	0.000
Time	14.105	0.000	Glucose	0.021	0.89
Glucose	0.725	0.40	Time	14.808	0.000
Time	13.899	0.001	Fructose	0.359	0.55
Fructose	0.123	0.73	Time	13.664	0.001
Time	13.933	0.001	Sucrose	0.001	0.98
Sucrose	0.087	0.77	Time	14.020	0.000

Time	14.034	0.000	D-fiber	0.719	0.40
D-fiber	0.007	0.93	Time	13.323	0.001
**Lactobacillus**					
Order	F-ratio	P-value	Order	F-ratio	P-value
Time	24.864	<.0001	Cho	5.048	0.029
Cho	1.908	0.17	Time	21.724	<.0001
Cho*time	7.224	0.010	time*Cho	7.224	0.010
Time	27.597	<.0001	sugar	14.604	0.000
sugar	9.058	0.004	Time	22.051	<.0001
sugar*time	6.903	0.011	Time*sugar	6.903	0.011
Time	28.350	<.0001	Glucose	13.990	0.001
Glucose	7.882	0.007	Time	22.242	<.0001
Glucose*time	9.961	0.003	Time*glucose	9.961	0.003
Time	26.157	<.0001	Fructose	12.551	0.001
Fructose	5.429	0.024	Time	19.035	<.0001
Fructose*time	6.932	0.011	Time*Fructose	6.932	0.011
Time	24.845	<.0001	Sucrose	8.560	0.005
Sucrose	6.645	0.013	Time	22.920	<.0001
Sucrose*time	2.415	0.13	Time*Sucrose	2.415	0.13
Time	23.896	<.0001	D-fiber	4.074	0.049
D-fiber	0.689	0.41	Time	20.511	<.0001
D-fiber*time	6.023	0.017	Time*fiber	6.023	0.017
**CHKCI001**					
Order	F-ratio	P-value	Order	F-ratio	P-value
Time	6.295	0.015	Cho	0.416	0.52
Cho	0.041	0.84	Time	5.919	0.018
Time	6.543	0.013	sugar	1.645	0.21
sugar	0.777	0.38	Time	5.670	0.021
Time	8.120	0.006	Glucose	9.468	0.003
Glucose	6.742	0.012	Time	5.394	0.024
Glucose*time	9.355	0.004	Time*glucose	9.355	0.004
Time	8.592	0.005	Fructose	12.221	0.001
Fructose	8.120	0.006	Time	4.491	0.039
Fructose*time	11.990	0.001	Time*Fructose	11.990	0.001
Time	6.285	0.015	Sucrose	0.280	0.60
Sucrose	0.124	0.73	Time	6.128	0.017
Time	6.636	0.013	D-fiber	2.271	0.14
D-fiber	0.800	0.38	Time	5.176	0.027
**Cutibacterium**					
Order	F-ratio	P-value	Order	F-ratio	P-value
Time	7.922	0.007	Cho	2.801	0.100
Cho	1.420	0.24	Time	6.541	0.013

Time	8.557	0.005	sugar	6.850	0.012
sugar	4.716	0.034	Time	6.424	0.014
Time	10.103	0.003	Glucose	12.283	0.001
Glucose	8.820	0.005	Time	6.641	0.013
Glucose*time	10.871	0.002	Time*glucose	10.871	0.002
Time	11.207	0.002	Fructose	17.327	0.000
Fructose	11.766	0.001	Time	5.646	0.021
Fructose*time	15.868	0.000	Time*Fructose	15.868	0.000
Time	7.822	0.007	Sucrose	2.946	0.092
Sucrose	2.314	0.13	Time	7.190	0.010
Time	7.766	0.007	D-fiber	2.326	0.13
D-fiber	0.740	0.007	Time	6.180	0.016
**Xanthomonas**					
Order	F-ratio	P-value	Order	F-ratio	P-value
Time	27.335	<.0001	Cho	3.457	0.069
Cho	0.891	0.35	Time	24.769	<.0001
Time	30.092	<.0001	sugar	8.472	0.005
sugar	4.196	0.046	Time	25.817	<.0001
sugar*time	6.328	0.015	Time*sugar	6.328	0.015
Time	28.863	<.0001	Glucose	7.579	0.008
Glucose	3.218	0.079	Time	24.502	<.0001
Glucose*time	4.709	0.035	Time*glucose	4.709	0.035
Time	27.526	<.0001	Fructose	7.067	0.010
Fructose	1.910	0.17	Time	22.369	<.0001
Time	28.710	<.0001	Sucrose	4.861	0.032
Sucrose	3.342	0.073	Time	27.191	<.0001
Sucrose*time	4.261	0.044	Time*Sucrose	4.261	0.044
Time	25.281	<.0001	D-fiber	1.163	0.286
D-fiber	0.031	0.86	Time	24.149	<.0001
**Enorma**					
Order	F-ratio	P-value	Order	F-ratio	P-value
Time	7.720	0.008	Cho	0.039	0.85
Cho	0.494	0.49	Time	8.174	0.006
Time	7.676	0.008	sugar	0.002	0.96
sugar	0.255	0.62	Time	7.927	0.007
Time	8.150	0.006	Glucose	0.629	0.43
Glucose	1.800	0.19	Time	9.321	0.004
Time	7.644	0.008	Fructose	0.261	0.61
Fructose	0.037	0.85	Time	7.419	0.009
Time	7.666	0.008	Sucrose	0.053	0.82
Sucrose	0.183	0.67	Time	7.796	0.007
Time	7.648	0.008	D-fiber	0.209	0.65

D-fiber	0.056	0.81	Time	7.496	0.008
**Staphylococcus**					
Order	F-ratio	P-value	Order	F-ratio	P-value
Time	7.692	0.008	Cho	1.368	0.25
Cho	0.472	0.50	Time	6.796	0.012
Time	7.758	0.007	sugar	1.732	0.19
sugar	0.768	0.39	Time	6.794	0.012
Time	11.329	0.001	Glucose	16.534	0.000
Glucose	12.293	0.001	Time	7.088	0.010
Glucose*time	17.091	0.000	Time*glucose	17.091	0.000
Time	13.167	0.001	Fructose	24.834	<.0001
Fructose	17.684	0.000	Time	6.017	0.018
Fructose*time	25.062	<.0001	Time*Fructose	25.062	<.0001
Time	7.335	0.009	Sucrose	0.063	0.80
Sucrose	0.003	0.95	Time	7.275	0.009
Time	8.766	0.005	D-fiber	7.028	0.011
D-fiber	3.910	0.053	Time	5.649	0.021
D-fiber*time	6.834	0.012	Time*fiber	6.834	0.012
**Rikenella**					
Order	F-ratio	P-value	Order	F-ratio	P-value
Time	5.975	0.018	Cho	0.185	0.67
Cho	0.000	1.0	Time	5.790	0.020
Time	6.140	0.016	sugar	0.955	0.33
sugar	0.340	0.56	Time	5.425	0.023
Time	6.098	0.017	Glucose	1.350	0.25
Glucose	0.517	0.48	Time	5.265	0.026
Time	6.066	0.017	Fructose	1.526	0.22
Fructose	0.404	0.53	Time	4.934	0.031
Time	6.028	0.017	Sucrose	0.218	0.64
Sucrose	0.086	0.77	Time	5.896	0.019
Time	6.015	0.018	D-fiber	0.943	0.34
D-fiber	0.140	0.71	Time	5.211	0.027

Table is read from left to right. Tested sequentially, fitting one additional effect at each step. First, we tested the effect of exercise (time) as the first sequential variable to see the strength of the association with a specific microbial taxon, and next we swap the order of exercise and the nutritional value to see if the nutritional variable has a significant association on its own, F value represents the strength of the association. Cho, Carbohydrates; D-fiber, Dietary fiber. *Statistically significant p value ($p \leq 0.05$).

Supplemental table S2. Characteristics of the exercise intervention in the leaner and the heavier twin groups at the first half and the second half of the exercise intervention [mean (95% CI)].

	**Heavier**		**Leaner**		**P-value**		
	Pre to mid	Mid to post	Pre to mid	Mid to post	Baseline	Time	Time*group
**n**	12	11	12	10			
**Male/female**	4/8	4/7	4/8	4/6			
**Strength workout total duration, min**	622.4 (506.2;738.6)	571.1 (449.3;692.9)	651.4 (527.5;775.2)	619.1 (481.1;757.2)	0.68	0.39	0.80
**Endurance workout 1 total duration, min**	598.6 (472.9;724.3)	721.3 (511.0;931.6)	658.7 (547.6;769.8)	780.0 (579.5;980.5)	0.28†	0.12	0.81
**HIIT workout total duration, min**	382.2 (285.4;479.0)	430.4 (297.8;563.0)	420.8 (330.6;510.9)	386.6 (277.4;495.8)	0.35†	0.82†	0.43†
**Endurance workout 2 total duration, min**	796.9 (493.7;1100.1)	790.8 (546.8;1034.8)	850.1 (709.2;991.04)	901.6 (723.8;1079.5)	0.75	0.75	0.59
**Strength workout average heartrate, Bpm**	110.6 (107.0;114.2)	109.8 (105.3;114.3)	113.6 (106.9;120.2)	115.0 (108.5;121.4)	0.26	0.93†	0.27†
**Endurance workout 1 average heartrate, Bpm**	126.1 (117.9;134.3)	124.2 (115.6;132.7)	133.9 (125.1;142.7)	132.1 (123.5;140.8)	0.032*	0.22†	0.85†
**HIIT workout average heartrate, Bpm**	130.5 (123.7;137.2)	128.1 (121.4;134.8)	134.4 (125.7;143.1)	133.6 (126.0;141.2)	0.17†	0.38	0.43
**Endurance workout 2 average heartrate, Bpm**	114.5 (110.5;118.4)	110.9 (106.2;115.7)	115.9 (111.0;120.8)	112.3 (106.8;117.8)	0.52	0.031*†	0.96†

P-value (linear mixed model) for Baseline: within-pair difference in the first half of the intervention, Time: first and second half difference in whole group, Time*group: did the training response differ within twin pairs. *Statistically significant p value (p≤0.05). †Logarithmic transformation.
